# Roadblocks of Urinary EV Biomarkers: Moving Toward the Clinic

**DOI:** 10.1002/jev2.70120

**Published:** 2025-07-17

**Authors:** Marvin Droste, Maija Puhka, Martin E. van Royen, Monica S. Y. Ng, Charles Blijdorp, Gloria Alvarez‐Llamas, Francesc E. Borràs, Anja K. Büscher, Benedetta Bussolati, James W. Dear, Juan M. Falcón‐Pérez, Bernd Giebel, Cristina Grange, Ewout J. Hoorn, Janne Leivo, Metka Lenassi, Alicia Llorente, Fabrice Lucien, Inge Mertens, Harald Mischak, Desmond Pink, Tobias Tertel, Swasti Tiwari, Dolores Di Vizio, Peter S. T. Yuen, Natasa Zarovni, Guido Jenster, Dylan Burger, Elena S. Martens‐Uzunova, Uta Erdbrügger

**Affiliations:** ^1^ Department II of Internal Medicine University of Cologne, Faculty of Medicine and University Hospital Cologne Cologne Germany; ^2^ Institute for Molecular Medicine Finland (FIMM) University of Helsinki Helsinki Finland; ^3^ Department of Pathology, Erasmus MC Cancer Institute Erasmus MC University Medical Center Rotterdam Rotterdam The Netherlands; ^4^ Kidney Health Service Royal Brisbane and Women's Hospital Herston Australia; ^5^ School of Clinical Medicine, Faculty of Medicine University of Queensland Herston Australia; ^6^ Institute of Molecular Biosciences University of Queensland St Lucia Australia; ^7^ Conjoint Internal Medicine Laboratory Chemical Pathology, Pathology Queensland Herston Australia; ^8^ Department of Internal Medicine, Division of Nephrology and Transplantation Erasmus MC University Medical Center Rotterdam Rotterdam The Netherlands; ^9^ IIS‐Fundación Jiménez Díaz‐UAM Madrid Spain; ^10^ RICORS2040 University Hospital Fundación Jiménez Díaz Madrid Spain; ^11^ REMAR‐IVECAT Group, “Germans Trias i Pujol” Health Science Research Institute and Nephrology Department “Germans Trias i Pujol” University Hospital Badalona Spain; ^12^ Department of Pediatrics II, Division of Pediatric Nephrology University Hospital Essen, University of Duisburg‐Essen Essen Germany; ^13^ Department of Molecular Biotechnology and Health Science University of Turin Turin Italy; ^14^ Centre for Cardiovascular Science, Queen's Medical Research Institute University of Edinburgh Edinburgh UK; ^15^ Exosomes Laboratory and Metabolomics Platform CIC bioGUNE‐BRTA, Bizkaia Science & Technology Park, Bilbao, Ikerbasque Research Foundation Bilbao Spain; ^16^ Institute of Transfusion Medicine, University Hospital Essen University of Duisburg‐Essen Essen Germany; ^17^ Department of Medical Sciences University of Turin Turin Italy; ^18^ Department of Life Technologies and InFLAMES Research Flagship University of Turku Turku Finland; ^19^ Institute of Biochemistry, Faculty of Medicine University of Ljubljana Ljubljana Slovenia; ^20^ Department of Molecular Cell Biology, Institute for Cancer Research Oslo University Hospital Oslo Norway; ^21^ Department for Mechanical Electronics and Chemical Engineering Oslo Metropolitan University Oslo Norway; ^22^ Department of Urology Mayo Clinic Rochester Minnesota USA; ^23^ Centre for Proteomics University of Antwerp and Health Unit, VITO Antwerp Belgium; ^24^ Mosaiques Diagnostics GmbH Hannover Germany; ^25^ Institute of Cardiovascular and Medical Sciences University of Glasgow Glasgow UK; ^26^ Nanostics Inc. Edmonton Alberta Canada; ^27^ Department of Molecular Medicine & Biotechnology Sanjay Gandhi Postgraduate Institute of Medical Sciences Lucknow India; ^28^ Department of Urology, Division of Cancer Biology and Therapeutics Cedars‐Sinai Medical Center Los Angeles California USA; ^29^ Renal Diagnostics and Therapeutic Unit, National Institute of Diabetes, Digestive and Kidney Disease NIH Bethesda USA; ^30^ NEXUS Project Day One srl Rome Italy; ^31^ RoseBio srl Milano Italy; ^32^ Erasmus MC Cancer Institute, Department of Urology, Laboratory of Experimental Urology Erasmus MC University Medical Center Rotterdam Rotterdam The Netherlands; ^33^ Kidney Research Centre, Ottawa Hospital Research Institute and Department of Cellular and Molecular Medicine University of Ottawa Ontario Canada; ^34^ Division of Nephrology University of Virginia Health System Charlottesville Virginia USA

**Keywords:** biomarkers, bladder, exosomes, extracellular vesicles, kidney, liquid biopsy, prostate, translation, urine

## Abstract

Despite remarkable interest in the biomarker potential of urinary extracellular vesicles (uEVs) and the identification of numerous promising candidates, their clinical translation still presents multiple challenges. The opportunities for successful translation are obvious, yet the main roadblocks on the way have hardly been systematically considered and more coordinated approaches are needed to overcome them. In the present review article, we have identified the most relevant roadblocks of clinical translation of urinary EV‐based biomarkers and discuss possible solutions to overcome them. These roadblocks are categorized as fundamental and technical but also related to development of novel biomarker assays and clinical acceptance. In addition, hurdles within the regulatory approval process are discussed. It is clear that various roadblocks to clinical translation of urinary EV biomarkers exist; however, they are addressable by promoting rigor and reproducibility as well as collaboration between basic and clinical scientists, clinicians, industry and regulatory bodies. Moreover, knowledge of obstacles for assay development and regulatory requirements should already be considered when developing a new biomarker to maximize the chance of successful translation. This review presents not only a status quo, but also a roadmap for the further development of the field.

## 1 Introduction

Extracellular vesicles (EVs) are submicron particles that transport various cargo between cells and organs. Given their versatile reported roles in diseases, EV research is considered to be a potential basis for the next frontier in precision medicine with a multitude of proposed clinical applications in diagnostics and therapeutics. After blood, urine is the second‐most studied human biofluid regarding EV and biomarker research (Erdbrügger et al. 2021). These urinary EVs (uEVs) are promising candidates for liquid biopsies since urine is available non‐invasively, in large quantities, and can even easily be collected at home (Erozenci et al. 2021).

uEVs may reflect pathophysiological processes of the organs in the urogenital tract and possibly other, more distant organs. In 2004, Pisitkun and colleagues demonstrated that uEVs contain protein signatures suitable for biomarker application (Pisitkun et al. 2004). Since then, the interest in leveraging uEVs for clinical diagnostics has grown exponentially with research focusing on urogenital cancers, acute and chronic kidney injury, kidney transplant rejection (Linxweiler and Junker 2020; Grange and Bussolati 2022), urinary tract infections (Mizutani et al. 2019), pregnancy and prenatal diagnostics (Zhou et al. 2020). Advances in nanoparticle analytics, methodological standardization, promising preclinical proof‐of‐concept data and significant financial investment brought the first EV‐based biomarkers to clinical trials (Tutrone et al. 2020; Kashani et al. 2017; Yu et al. 2021; McKiernan et al. 2016). Of note, the projected global EV market size in 2026 is expected to reach $512.6 million (Dash et al. 2023). However, to our knowledge only one uEV biomarker has been integrated into diagnostic and therapeutic workflows demonstrating a clear gap from benchtop results to clinical utilization (Tutrone et al. 2020).

To promote rigor, reproducibility and standardization in this flourishing, yet increasingly complex research field, the International Society for Extracellular Vesicles (ISEV) founded the Urine Task Force. A comprehensive position paper on uEV state‐of‐the‐art research (Erdbrügger et al. 2021) was published and a Quick Reference Card on pre‐analytical processing and storage of uEVs (van Royen et al. 2023) was developed to guide and advance the utilization of uEVs as diagnostic tools. The next step is to identify the roadblocks that prevent clinical translation to realize the full potential of uEV biomarkers. In this review, we identify, describe and propose solutions to the critical roadblocks to clinical translation of uEV biomarkers.

## 2 Defining Roadblocks of Research That Hinder Clinical Translation of uEVs

**FIGURE 1:**
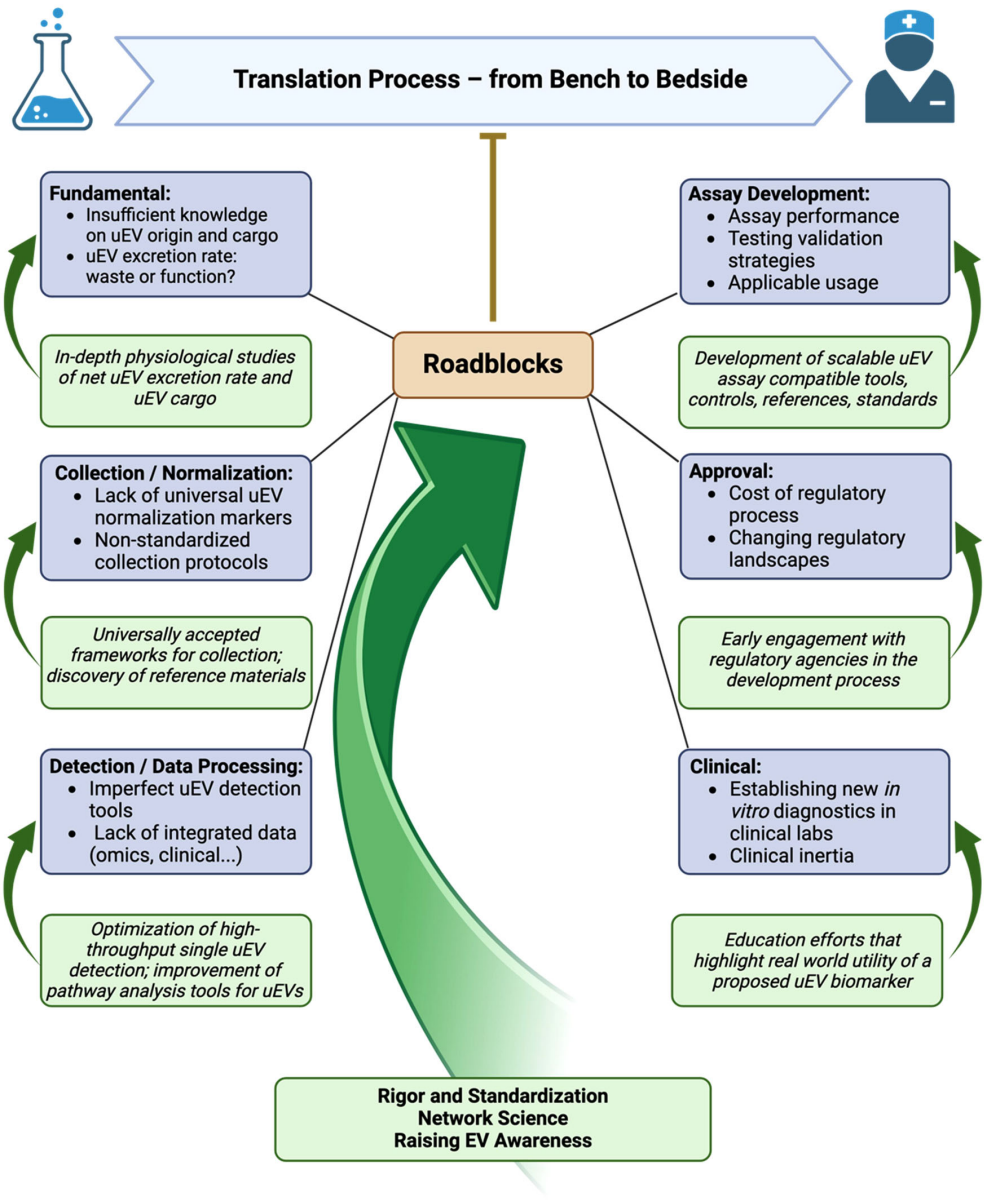
Summary of the translational process of uEV biomarkers, roadblocks on the way and potential solutions for overcoming them. Figure created using BioRender.com.

We define a **roadblock** as an entity or an (in)action that blocks or severely hinders the EV community from making progress. We divide roadblocks into four categories: fundamental, methodological, approval (legal) and clinical. In general, these roadblocks result from physical and technical limitations, unknown variables, but also lack of expertise or awareness as well as lack of resources of any kind. We summarize and graphically depict the most important identified roadblocks and the possible strategies to overcome them in Table 1 and Figure 1.

**TABLE 1:** Main roadblocks in the translation of uEV biomarkers to clinics.

**Roadblock** What is keeping us from moving forward?	**Solutions** How to overcome these obstacles?	**Impact** How will the field move forward once the roadblock is removed?
**Fundamental roadblocks**		
**Insufficient knowledge on uEV origin and cargo composition in health and disease** Contributions from different organs and physiological as well as pathological processes influence the uEV pool. This could include variable levels of EVs from the male and female reproductive tract, but also interpersonal variability with regards to circadian rhythm, diet, lifestyle and so forth.	In‐depth physiologic studies of uEV excretion and factors affecting it. Studies needed in humans and animals.	Identifying the uEV carrier of a biomarker of interest enables more precise isolation and capture of relevant EVs, improving assay precision and enhancing biomarker performance.
**Limited understanding of uEV excretion rate** as net result of secretion and uptake and factors influencing this rate.	In‐depth physiologic studies of uEV secretion and uptake as above, ideally utilizing endogenously labelled uEVs.	Knowledge of these factors can optimize uEV quantification for biomarker discovery, but also lead to more mechanistic insight.
**Limited knowledge on function of EV biosynthesis, secretion and their cargo in health and disease**. Not knowing if the uEV cargo is waste or has a function, or both.	Develop tools and readout systems (e.g., uEV detection assays, model systems, genetic and small molecule screens) to manipulate and functionally study uEV production, secretion and cargo.	Understanding why certain changes occur results in better disease biomarkers and could be the basis for therapeutic interventions, since uEV analyses provide a chance to identify new pathways that cannot easily be ascertained through tissue studies.
**Methodological roadblocks**		
***Collection***		
**Limited or no access to preanalytical information**.	Collect biobank data using standardized documentation and fulfil preanalytical reporting requirements as outlined by MISEV, EV‐TRACK and regulatory bodies. Apply SPREC codes and ensure inclusion of all relevant regulatory reporting parameters.	Identification of critical parameters that influence study outcome. Improved (future) study design and execution. Increased rate of reproducibility by comparing samples with similar SPREC codes.
**Lack of synchronization of collection, storage and quality control** (guidelines, SOPs).	Establish and implement a validated convention/framework for urine and uEV collection, storage and quality control.	Reduced technical variation of experimental results. Increased quality of validation studies and inter‐laboratory studies.
***Normalization***		
**Natural intra‐ and inter‐individual biological variability of urine composition and concentration**.	Identify surrogate measurement parameters and/or biomolecules that allow quantitation of uEVs.	Accurate quantitation of uEV molecules will allow normalization across multiple samples and studies and facilitate uEV‐biomarker discovery and validation.
**Lack of housekeeping genes, proteins, metabolites and other reference biomolecules** in specific diseases.	Discover and validate biomarkers in specific preanalytical and disease conditions.	Disease‐specific validation of reference biomolecules will increase chances of finding relevant biomarkers and increase reliability of these biomarkers and will decrease chance of biases.
**Relying on urine creatinine** as an easy and reliable correction marker without recognizing its variability in health and disease.	Studies validating the use of urine creatinine for normalization in various diseases, which can be easily combined with well set‐up biomarker studies.	Creatinine is easily available, and if its use for normalization holds through‐out the field it can be used where other normalization are not available, or unstable.
**Lack of stable/validated tissue‐specific markers for normalization**.	Studies relating the abundance of tissue‐specific biomarkers in uEVs with their abundance within the tissue itself.	Validated tissue‐specific biomarkers would help correcting for altered uEV release by tissues, for example, because of hypertrophy or loss of the tissue, or because of change in ratio prostatic fluid versus urine.
***Detection technology***		
**Heterogeneous nature of uEVs**.	Analysis of individual uEVs or efficient isolation/sorting of different uEV subpopulations.	Better identification of uEV subpopulations (e.g., uEV types, uEV origin).
**Heterogeneous composition** of clinical urine samples.	Standardized and high‐quality isolation and purification protocols with limited loss of EV material or highly uEV specific detection strategies.	Robust and specific detection of uEVs and uEV subpopulations.
**Lack of generic markers that are present on all uEVs**.	Identification of generic EV surface markers or highly uEV specific labelling strategies.	Reliable detection of uEVs in complex clinical samples.
**Lack of detectable and validated disease specific uEV (surface) markers**.	Large studies to identify disease specific uEV (surface) markers. Development of highly sensitive technologies to detect these probably scarce markers.	uEV based assays to diagnose and monitor disease.
**Scalability** of sensitive uEV detection.	Technological developments toward higher throughput and automation of uEV detection and analysis.	Development of uEV assays for large‐scale diagnostic use.
**Lack of good (cross technology) calibrator and reference materials**.	Development of high‐quality and well characterized uEV mimics.	Reliable and reproducible uEV analysis and interpretation of uEV data between technologies and laboratories.
**Lack of cross‐technology consensus** of uEV analysis.	Cross‐technology comparative studies using high‐quality and well characterized uEV reference material.	Reliable and reproducible uEV analysis and interpretation of uEV data between technologies and laboratories.
**Data processing**		
**EV (omics) data integration is complex** for datasets with different EV collection and processing procedures .	Studies which use multimodal datasets from the same samples and utilize the appropriate computer models for data integration including use of artificial intelligence.	Improved reliability and statistical power of knowledge extracted from integrated datasets.
**EVs may not be a miniature representation of the parent cell**: presence and processing of RNAs, proteins and metabolites in EVs is fundamentally different from the living parent cell.	Develop pathway and cell type profiles for interpretation of EV omics data. Adapt pathway analysis software for EV data interpretation.	EV omics data can be used to determine expression and functional correlations and resolve the EV‐origin.
**Assay development roadblocks**		
**Applicable usage areas** Lack of knowledge about best or non‐applicable usage areas results in poorly focused studies for assay development.	Perform broad testing with varied samples from appropriate biorepositories already at the assay development phase.	Elucidation of the best areas of use earlier in development helps to define the exact clinical use and enables development of assays more efficiently.
**Testing preanalytical practices** Due to lack of standard uEV workflows, applicable preanalytical practices need to be tested separately for each assay, which is slow and expensive.	Develop general or assay‐type specific recommendations and guidelines of preanalytical workflows. As more uEV assays will be developed, the most successful practices can be adopted for broader use.	Assay development will be faster as adherence to the guidelines saves time from testing preanalytical workflows.
**Managing performance tests and validation** Particularly academic researchers have often missing or sparse knowledge and capacity to manage the performance tests and validation. Performance requirements differ for IVD and LDT approval.	Education to academic researchers, new channels to foster collaborations and access to proper infrastructures for high‐capacity testing and consulting by regulatory authorities who have experience with urine or uEV biomarker assays, IVD and LDT requirements.	More biomarker candidates can take a faster path through performance tests and validation. Establishing and reporting performance characteristics according to FDA, CLIA, ISO or other standards (state regulations, laboratory accreditation program standards) enables and expedites the launch of the assays for clinical use.
**Approval roadblocks**		
**Cost of regulatory process** Development of new in vitro diagnostics are subject to extensive regulatory oversight in order to protect patients.	Consider focusing on LDT or in house IVD as a first step to establishment of the assay.	Lower process for regulatory clearance. However, this may limit widespread adoption.
**Changing regulatory landscape** The processes for regulatory approval can change over time.	Engage with regulatory agencies early in the development process to learn of any pending changes to regulatory approval processes.	Approval is not impacted by sudden changes in regulatory requirements.
**Clinical roadblocks**		
**Establishing new in vitro diagnostics in clinical labs** Excess costs, training, or the need for specialized equipment may impede adoption in clinical laboratories.	Developing an LDT or in house IVD allows for a single centralized laboratory to maintain necessary infrastructure and train staff.	Overcomes barriers to implementation at the single lab level. However, this may not be applicable for point‐of‐care tests.
**Clinical inertia** Clinicians may be reluctant to employ new tests due to concerns about superiority versus current tests or about impact on workflow.	Education efforts that highlight real world utility.	Clinicians recognize the value of a new test and its clinical utility and gradually expand usage.
**Downstream commercialization** in a rapidly changing clinical landscape.	Expect to conduct multiple appropriate clinical studies demonstrating how the test fills diagnostic gaps. Early partnership with clinical scientists.	Ensuring that the developed test retains its clinical relevance and is therefore of continued interest for physicians.

Abbreviations: CLIA, Clinical Laboratory Improvement Amendments; EVs, extracellular vesicles; FDA, Food and Drug Administration; IH‐IVD, in house‐in vitro diagnostic; IVD, in vitro diagnostic; ISO, International Organisation for Standardisation; LDT, laboratory developed test; MISEV, Minimal Information for Studies of Extracellular Vesicles; RNA, ribonucleic acid; uEV, urinary extracellular vesicles.

The figure comprises the most relevant hurdles in each step of the translation process and highlights the overall importance of rigor and standardization as well as networking within the field for successful clinical adoption of uEV‐based biomarkers. See Table 1 for details. Figure created using BioRender.com.

## 3 Fundamental Roadblocks

**FIGURE 2:**
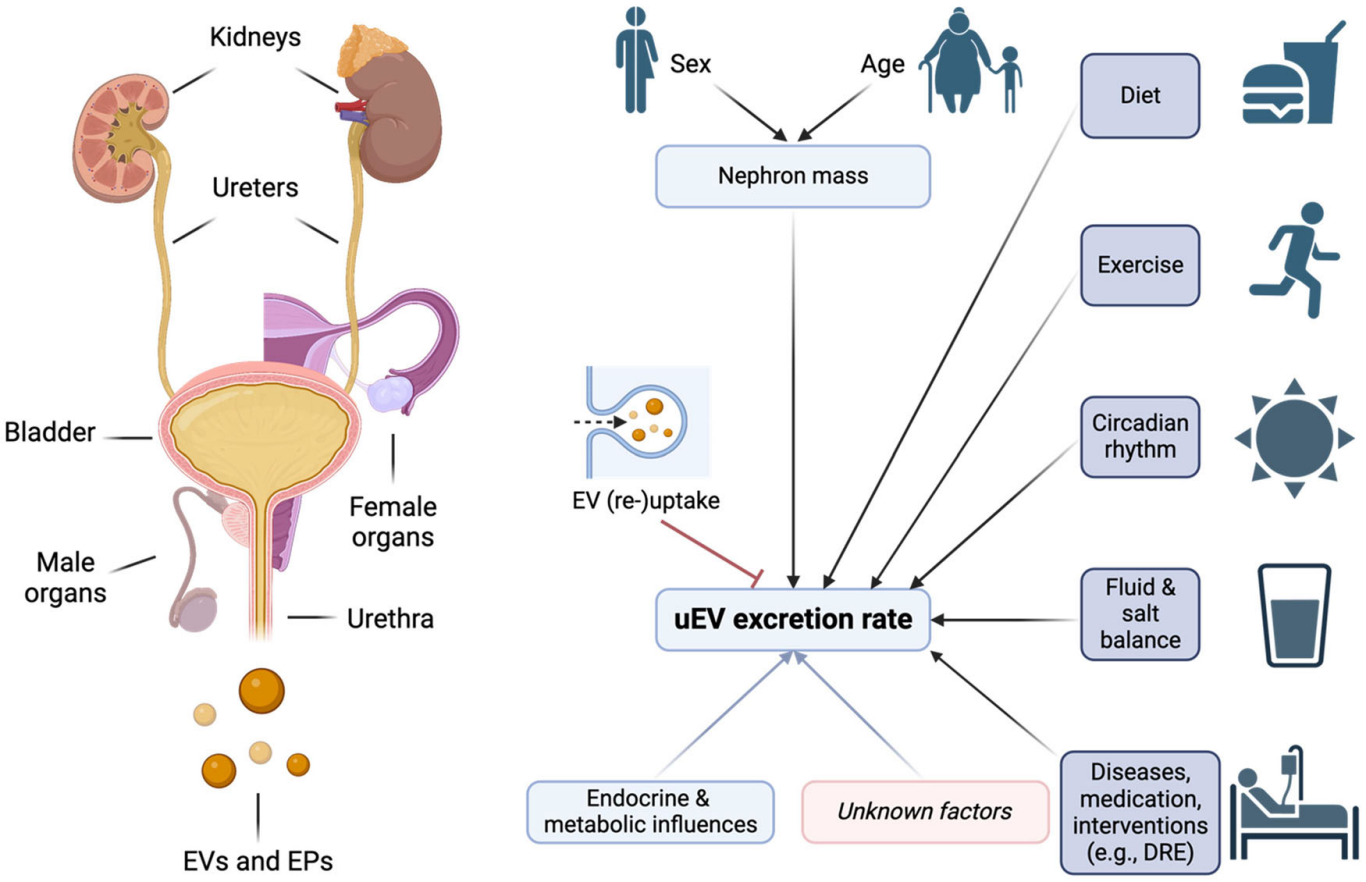
Schematic overview of the factors determining urinary extracellular vesicle (uEV) excretion. uEV excretion is the net result of renal EV secretion minus uptake plus contributions from other organs of the genitourinary system. The kidney is the main source of uEVs and therefore nephron mass is an important determinant of the uEV excretion rate. Nephron mass, in turn, is determined by sex and age—men generally have larger kidneys and nephron number decreases with age. Other factors determining uEV secretion are circadian rhythm, dietary intake, medication, water and salt balance and exercise. Although it is clear that cells can take up EVs, the factors influencing this process are less well understood. EVs, extracellular vesicles; uEVs, urinary extracellular vesicles; EPs, extracellular particles; DRE, digital rectal examination. Figure created using BioRender.com.

A full assessment of the potential of uEVs as a source of next‐generation biomarkers warrants the consideration of several fundamental aspects including the process of urine production, the physiology of urinary tract organs and their function, as well as the biology of EVs. The majority of the uEV pool consists of EVs from the kidney, ureters, bladder, urethra and the male or female reproductive organs. The male reproductive system secretes its fluid from the testes, epididymis, seminal vesicles and prostate in the urethra, while the uterus, ovaries, fallopian tubes, cervix and vagina constitute the secretome of the female reproductive system. The kidney is one of the main sources of uEVs (Nørgård et al. 2022; Barreiro et al. 2023; Dwivedi et al. 2023) and nephron mass seems to determine the uEV excretion rate (Blijdorp et al. 2022). The nephrons, which are the functional units of the kidney, work through a two‐step process: the glomerulus filters blood and the tubule returns needed substances to blood and removes waste. As a result, urine constituents are the net product of glomerular filtration plus tubular secretion and contributions from other urogenital organs minus reabsorption. Consequently, it is important to identify factors that influence EV secretion and uptake to correctly interpret the net uEV excretion rate. Although normalization strategies are regularly used to carry out biomarker quantification without knowing the uEV excretion rate, a ‘perfect’ normalizer for uEVs has not been established. Understanding the true secretion rate of different uEVs and their subpopulations and the factors which influence it could help to establish new normalization strategies that in turn may unveil novel biomarkers with increased sensitivity and specificity. The current (limited) literature supports that cellular EV secretion into the urine depends on circadian rhythm, dietary intake, medication, water and salt balance, exercise, as well as manipulations of the urogenital tract during diagnostic or therapeutic procedures (for instance, digital‐rectal examination or cystoscopy) (Castagna et al. 2015; Koritzinsky et al. 2019; Qi et al. 2016; Park and Moon 2022) (Figure 2). Urine void timing is also important as, for instance, first morning urine is generally more concentrated due to less water intake during the night.

The overall EV secretion rate depends on the total number of cells secreting EVs into the urine (Barreiro et al. 2023). Chronic kidney disease is usually accompanied by nephron loss and will therefore reduce the uEV excretion rate (Blijdorp et al. 2022). Similarly, current literature supports that uEV excretion rate is sex and age dependent, likely due to differences in nephron mass (Barreiro et al. 2023, Qi et al. 2016). In males, the contribution of prostate‐derived EVs may also increase with age as many men will develop benign prostate hyperplasia over time (Kim et al. 2022).

The effect of EV uptake on the uEV excretion rate is less clear as EV uptake routes are not yet fully characterized. It has been suggested that the hormone vasopressin regulates the uptake of EVs in the kidney (Oosthuyzen et al. 2016). Further delineating the process of EV uptake is warranted and will offer the opportunity to study the functional roles of uEVs (e.g., as intercellular or intranephron messengers) and to explore uEVs as therapeutic tool (Grange and Bussolati 2022; Huang et al. 2022). Establishing the uEV secretion and uptake rates is relevant because physiological or disease‐related factors can change either one or both of them as well as the subpopulations and compositions of the uEVs (Wu et al. 2021).

Understanding the effects of physiological or disease‐related factors on uEV excretion rate, and/or uEV composition is essential for the accurate interpretation of uEV biomarkers in the clinical context. A biomarker may demonstrate clinical value even if its underlying mechanisms are not fully understood. However, uncertainty of the biomarker's physiological role can lead to two major issues: the selection of suboptimal uEV enrichment strategies, potentially compromising assay performance, and the risk of drawing inaccurate clinical conclusions. Even if all modifying factors are well‐controlled or monitored, EVs do not always qualitatively and/or quantitatively reflect the content in the cells of origin. Although human kidney and uEV transcriptomes have shown similarities overall and particularly for the kidney‐enriched genes (Dwivedi et al. 2023), a large‐scale proteomics study in a homogeneous group of experimental animals showed that kidney‐to‐uEV correlations are formally stronger for transmembrane proteins than for cytosolic proteins (Wu et al. 2021). To deepen our understanding, it is essential to establish a comprehensive inventory of RNA transcripts and proteins preferentially secreted via EVs and to gain better insight in the processes that select and sort them. From a mechanistic point of view, the EV biogenesis pathway significantly impacts EV cargo, which is different for various EV subpopulations (Buzas 2023). Additionally, external factors that influence genitourinary tract physiology can also affect EV cargo composition.

The fundamental roadblocks described in this section are inherent to the fact that EVs were discovered relatively recently. One can also argue that our technological limitations can be counted as fundamental. With higher resolution and precision technology EV biology can be better dissected. However, with technological advances enabling single EV analytics and in vivo models with endogenously labelled cell‐type specific EVs, we should be able to further clarify the role of EVs in health and disease.

## 4 Methodological Roadblocks

**FIGURE 3:**
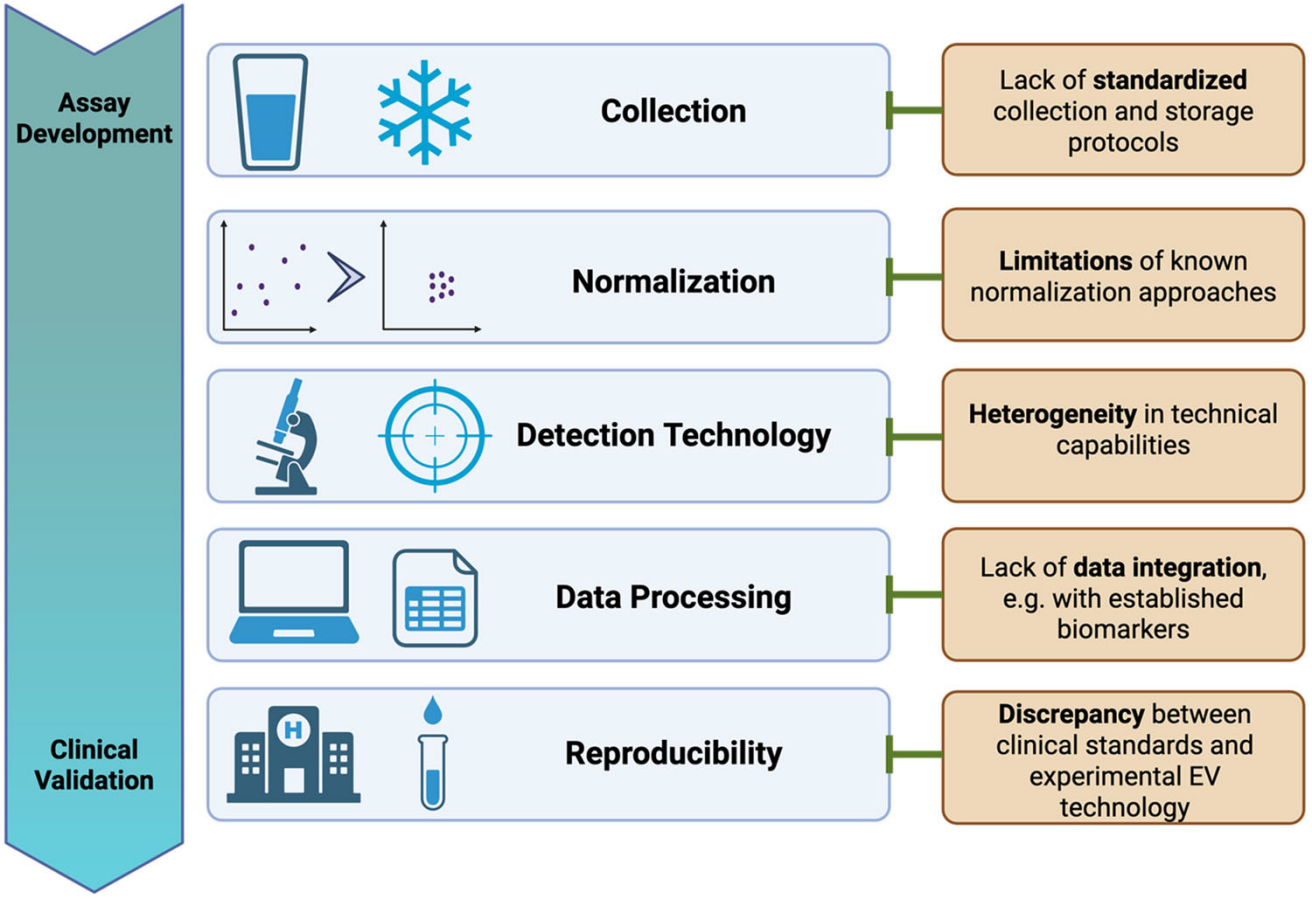
Overview of methodological roadblocks during assay development and clinical validation of uEV‐based biomarkers. Challenges in development of a biomarker product arise from both non‐standardized collection and storage protocols, as well as imperfections of normalization strategies. During further clinical validation, insufficient precision of novel, non‐established EV detection technologies, lack of data integration due to unavailability of well‐curated clinical datasets and discrepancy between clinical standards and experimental EV technology can be problematic. Figure created using BioRender.com.

Methodological roadblocks of clinical translation of uEVs include difficulties with standardized urine collection and processing, lack of quality control, the lack of appropriate normalization and quality control strategies for urine and uEV biomarkers, the technically challenging development of reproducible uEV separation and detection technologies, as well as the processing and integration of big uEV data, which requires specialized computational tools and extensive expertise (Figure 3).

### 4.1 Collection

Reproducibility is an essential requirement for the effective clinical translation of uEV research. Due to the variable nature of urine as a biofluid, uEV studies are particularly sensitive to variables at the beginning of the research ‘chain’ including patient selection, sample collection, type of timed urine collection (e.g., 24‐h vs. spot urine) and data normalization. However, studies on collection, pre‐analytical processing and pre‐analytical storage of uEVs are limited (Erozenci et al. 2021; Zhou et al. 2006; Barreiro et al. 2021). A recent survey by the Spanish Society for research and innovation in Extracellular Vesicles (GEIVEX) compared practices in 43 laboratories including seven working on uEVs (López‐Guerrero et al. 2023). The interrogation of seven Standard Pre‐analytical Code variables (SPREC) demonstrated strong variability and insufficient overlap in the protocols for collection, pre‐analytical processing and pre‐analytical storage (López‐Guerrero et al. 2023; Betsou et al. 2010). Moreover, in some cases researchers had no access to all relevant pre‐analytical sample information. Such results indicate that the scarce number of studies addressing the pre‐analytical handling of uEVs and the subsequent lack of synchronized Standard Operating Procedures (SOPs) stand as major roadblocks toward the clinical translation of uEV research.

To address the specific characteristics and challenges of urine as a source of EVs, the ISEV Urine Task Force published a position paper that summarizes the current best practices in uEV research and provides guidelines for improved rigor, reproducibility, inter‐operability and minimal reporting of multiple aspects in the collection, handling and storage of urine samples and uEVs (Erdbrügger et al. 2021). To facilitate the implementation of the best practices and to provide practical guidelines for their application in daily research and clinical laboratories, recommendations on storage parameters from the position paper were published in the form of a Quick Reference Card addressing the six critical stages preceding uEV analysis, that is, biobanking, storage of urine prior to processing, preprocessing, storage, defrosting and transportation of urinary supernatant or uEVs (van Royen et al. 2023).

Despite encouraging steps toward rigorous pre‐analytical practices in uEV research such as the introduction of SPREC and storage guidelines, significantly more work needs to be done to fully unfold the translational potential of uEVs. The likely influence of home collection variables, particularly in future large‐scale studies or even population screening programs, would pose additional levels of variability to an already diverse landscape of laboratory practices. Synchronization of SOPs, integration of currently existing tools like MISEV (Théry et al. 2018), EV‐TRACK (Van Deun et al. 2017), SPREC (Betsou et al. 2010) and evaluation of the most critical collection, preprocessing and storage parameters by dedicated research efforts and funding are a necessary prerequisite that can minimize the effect of pre‐analytical variability on research output and reproducibility, and so facilitating future clinical translation. It is also critical for researchers to establish and implement a collaboratively validated framework for urine and uEV collection and storage, as exemplified by the recently published MIBlood‐EV framework, which has addressed this need in blood‐derived EV analytics (Lucien et al. 2023). This will lead to improved reproducibility of study designs and hence reduce the technical variation in experimental results to increase the quality and performance of validation studies.

### 4.2 Normalization

Contrary to EVs circulating in blood, the concentration of uEVs is not directly dependent on their excretion along the urogenital tract, but it is influenced by the large differences in urinary concentration during the day (Sands and Layton 2014; Salih et al. 2016). In fact, urine is one of the most dynamic biofluids for EV analysis (Taylor and Curhan 2008). Although 24‐h urine collections are often used in the clinic to allow normalization of certain analytes by their excreted amount within a given period, these timed collections are not always reliable, due to inconvenience, patient errors and uncertainties about EV integrity after inappropriate storage (Oosthuyzen et al. 2013; Armstrong et al. 2018; Boyd et al. 2018). The prerequisite to a widespread clinical application is the ability to accurately measure uEV‐derived biomarkers in random spot urine samples. Efforts are underway to discover and validate broadly applicable uEV reference (housekeeping) genes and proteins across different preanalytical and disease conditions (Barreiro et al. 2021; Pinheiro et al. 2024). For simple clinically applicable workflows, simultaneous reference measurements should be included in the EV biomarker testing. Putative pan‐uEV markers may have limitations because their expression may differ between segments of the urogenital tract (Limbutara et al. 2020; Blijdorp et al. 2021). A **(disease‐)specific marker** ratio is preferable, especially if it has a (patho‐)physiological rationale. However, such markers need to be developed separately in each biomarker and disease setting (Erdbrügger et al. 2021). This is exemplified by two uEV mRNA tests (i.e., for prostate cancer and for kidney transplant rejection), which were developed on the same platform but utilize completely different reference genes (McKiernan et al. 2016, El Fekih et al. 2025). **Creatinine** is a simple option to normalize uEV concentration, its excretion rate being independent of urine concentration (Blijdorp et al. 2021). Urine creatinine concentrations would need to be measured in addition to the EV cargo analysis in the same sample. Nevertheless, urine creatinine measurements are only reliable when measured in a steady state condition, and not during acute changes in kidney function (glomerular filtration rate) (Waikar et al. 2010). In addition, the production of creatinine is determined by the body skeletal muscle mass, leading to overestimation of uEV excretion in women versus men (Blijdorp et al. 2022). The biological sex bias by muscle mass and nephron number can partially balance each other out (Blijdorp et al. 2022), but this should be validated in larger studies. A way to overcome this roadblock would be to develop semi‐generalizable EV markers or housekeeping markers, such as proteins constitutively excreted by specific nephron segments, the bladder, or the prostate, which could—as a denominator—normalize biomarkers to the uEV excretion rate of that specific tissue, organ segment, or organ.

In the case of prostate‐derived EVs, prostate‐specific proteins and mRNAs are typically utilized for normalization. The concentration of the prostate‐specific antigen (PSA) protein in urine and EV‐mRNAs from kallikrein related peptidase 3 (KLK3, the gene encoding PSA) or SAM pointed domain containing ETS transcription factor (SPDEF) have been proposed as an index for the amount of prostate fluid and prostate‐EVs in urine (Iwakiri et al. 1993; McKiernan et al. 2020; Hendriks et al. 2016). Ideally, such normalization markers would be defined for all organs (bladder, uterus, etc.) and cells contributing to the uEV pool. However, the expression of such housekeeping markers needs to be studied in health and disease. Alternatively, co‐localizing biomarkers, especially those changing in opposite directions, could be identified for specific applications.

### 4.3 Detection Technology

A plentitude of highly advanced technologies, including omics approaches, are regularly used for the discovery of uEV biomarkers. Yet, the clinical translation of routinely applicable uEV detection technologies is still in its infancy. For ideal integration in clinical workflows, uEVs should be analysed in minimally processed urine samples to avoid loss of material during isolation steps and bias toward specific uEV populations, but also to keep analysis time short enough for integration into clinical workflows (Wu et al. 2023). Major challenges in EV analysis arise from their small size and heterogeneous nature in terms of size, structure and composition. This phenotypic complexity, together with the heterogeneous composition of biofluids, makes it difficult to establish the exact physiological functions, cargo or origins of EVs, which may limit their clinical applicability. In the past, the majority of studies applied ‘bulk’ analyses of the total EV fraction, which typically utilize established methods such as immunoblotting, ELISA or PCR to study the EV sample as a whole. Although these ‘bulk’ analyses of the EV sample typically do not require extensive pre‐processing of the sample and often match the required time of analysis, they do not address the heterogeneity in EVs and their complex biofluid environment. Of note, some EV biomarker signatures can be found with bulk analysis (Tutrone et al. 2020) as it has been done successfully in prostate cancer. Thus, as long as an analyte is abundant and specific (such as a highly expressed RNA in a certain type of cancer), the advantages of bulk analyses (shorter analysis time, less specialized equipment and training) can and should be fully exploited to increase the translational potential. However, ‘single EV’ analysis (or at least subpopulation‐enriched analysis) is essential to allow selective studies of specific EV subpopulations originating from organs or diseases of interest within the mixed composition of urine (Bordanaba‐Florit et al. 2021). Technologies offering single EV resolution for large EV quantities (often through nanotechnological modification of methods such as flow cytometry or fluorescence microscopy) provide insights into how many individual EVs carry a specific biomarker, thus enabling the accurate analysis of biomarker‐positive subpopulations. A significant advantage over bulk EV analysis in the context of biomarker studies is the opportunity to increase sensitivity and specificity with regard to low or scarcely expressed biomarkers or EVs originating from less abundant tissues. Multiplex analyses on the single EV level contribute to this advantage (Cai et al. 2024). It can also be hypothesized that this increase in sensitivity and specificity translates into a higher diagnostic accuracy in early disease stages, for instance in asymptomatic patients or in screening scenarios. Nevertheless, it should be noted that some classical ‘bulk’ EV analysis strategies, such as ELISA‐like approaches, targeted proteomics and immunoblotting methods, can be modified for instance by including specific capture and detection antibody pairs to increase specificity toward uEV subpopulations (Duijvesz et al. 2015; Takizawa et al. 2023; Kawakami et al. 2021).

Although some of the currently available technologies aim to detect individual EVs without an exogenous label, the majority of single as well as bulk EV analysis tools still depend on a label for detection. Typically, generic fluorescent labelling of luminal or membrane compartments is applied (Simonsen 2019). Although signals derived from these generic EV labels are generally bright enough for efficient detection of larger EVs in most experimental settings, the capacity of small EV detection differs notably among the available devices, resulting in a bias toward larger EVs. Moreover, the dye‐specific behaviour and potential background levels emphasize the need for optimization if EV‐labelling strategies for different platforms and the importance of proper dye‐only controls (Simonsen 2019; Fortunato et al. 2021). Altogether, the lack of a widely accepted, universal but EV‐specific labelling approach limits EV analysis of minimally processed urine. This roadblock has been discussed in more detail in the section ‘normalization’ and will therefore not be elaborated here.

The quantitation of uEV population as a whole (i.e., the ‘total’ uEV count in a given sample) will be most likely insufficient to utilize it with diagnostic potential, as it may not reflect disease processes. For example, it is possible that in state of disease the total number of EVs does not change but their cargo content does. Therefore, it may be necessary to detect proven and reliable disease‐specific uEV‐based biomarkers instead (Junker et al. 2016; Khoo et al. 2024). Despite a tremendous global effort, the number of reliable disease‐specific uEV (surface) markers is limited. This may be in part due to the fact that various diagnostic uEV markers are expressed in rather low amounts, which hinders their detection, especially due to the difficulty in discriminating the marker signals from background signals in numerous detection methods (Ferguson et al. 2022).

Despite the challenges that arise when aiming at sufficient sensitivity in single EV analysis, detection of individual uEVs should not be seen always as a roadblock by itself. Several technologies that allow the analysis of particles much smaller than EVs have been recently developed within as well as outside of the EV research field, including molecular microscopy approaches, atomic force microscopy and electron microscopy. Sequencing and PCR technologies that are capable of analysing extremely low quantities of nucleic acids can also be adopted for EV analysis (Bordanaba‐Florit et al. 2021). However, despite the notable effort by the EV community within the last decade, these developments have not yet contributed to clinical translation of uEV products due to the limited scalability and/or the need of specialized technology and skills.

The challenges presented by the previously discussed limitations in sensitivity and specificity are further aggravated by the lack of consensus on standardized reference materials for calibration of equipment, protocols and assays, which also affects the comparability of results between different laboratories (Geeurickx et al. 2019; Valkonen et al. 2017; Welsh et al. 2020). Especially in quantitative assays, definition of ‘negative/positive’ cutoffs is virtually impossible without measuring standard samples. Moreover, such reference materials are crucial for quality control purposes in all clinical laboratories. Thus, a detection technology or assay without readily available standard materials will likely fail accreditation in the carefully controlled clinical diagnostics workflow. To address this issue, several groups have applied synthetic nanoparticles for calibration, although their fluorescence properties and refractive index typically do not resemble those of natural EVs (Welsh et al. 2020; Deumer et al. 2024). Of note, given the heterogeneity of EV subpopulations, it is virtually impossible to create synthetic reference materials that reflect all properties of the entire EV composition derived from a given biofluid. To overcome this, there are increasing efforts to generate biological membrane vesicles as ‘EV mimetics’ for calibration purposes (Lozano‐Andrés et al. 2019), and moreover, it has been proposed to apply ‘standard donor urine samples’ for calibration, especially in technical studies comparing different detection technologies (Droste et al. 2021). The real‐world use of such an approach is, however, still very limited, since large amounts of the standard donor samples would be necessary, and degradation of samples during storage resulting in insufficient consistency cannot be excluded.

Altogether, the high inter‐laboratory variability in EV analysis, the lack of standardization in sample pre‐processing, measurement protocols, equipment and its calibration, data processing and interpretation of the results all interfere with the clinical potential of uEVs. However, a test developer controlling as many variables as possible, can still produce reliable data and find novel EV signatures as biomarkers which can pass regulatory checks as seen with an already successful uEV biomarker (Tutrone et al. 2020).

### 4.4 Data Analysis and Integration

Omics data, such as genomics, transcriptomics, proteomics and metabolomics, provide layers of information that characterize a biosystem from a biomolecular perspective (Haukaas et al. 2017). As EVs are relatively complex carriers of cargo and signals, their functions and dysregulation might be best revealed using (multi‐)omics technologies. Indeed, recent research examples demonstrate the potential of (multi‐)omic approaches to identify specific disease signatures or unique druggable targets. An orthogonal, multi‐omic strategy may provide more context to a potential biomarker especially when the biomarker measurements are collected and interpreted in alignment with the clinical features of the patient (age, gender, medication regime) (Clos‐Garcia et al. 2018). However, challenges remain as multi‐omics require specialized analysis frameworks and skills, are relatively expensive, and time consuming—making them inaccessible for small research laboratories and companies.

An additional issue for the (urinary) EV field is that EV (omics) datasets are generated with different EV collection and processing procedures for the different types of omics and across different research groups. For proteomics, high EV purity is essential to eliminate abundant urine non‐EV contaminants (such as high molecular weight Tamm–Horsfall protein (THP or UMOD)), while for transcriptomics, easier high‐yield EV isolation methods combined with RNase treatment might be implemented to eliminate extravesicular transcripts. Changes in the separation and processing methodologies do result in analyses of overlapping but not identical EV subpopulations. Often, omics data integration is performed separately from the main‐stream individual omics data analyses without synchronizing the protocols, planning and structure of the whole experiment. Data integration is most complex and noisy for EV (omics) data derived from different independent datasets (Jendoubi 2021).

One question in data design is how to obtain samples for multimodal analysis, for example, repetition over time, splitting of material, or biological replicates (Cavill et al. 2016). Modalities can be horizontal (same type of omics with different samples, leading to a meta‐analysis) or vertical (different types of omics), which is more closely aligned with the general idea of integration (Richardson et al. 2016). Depending on these premises, there is a choice of whether to combine data (i) before analysis, (ii) after performing a step for variable reduction or (iii) integrate models to generate a high‐level model. Furthermore, the aim of integration can range from (i) adding supplementary information to the initial analysis, (ii) gaining insight into the underlying biology of the process or (iii) enhancing the predictive capabilities of the model (Hirai et al. 2005; Safo et al. 2018; Smolinska et al. 2012). An example of a pipeline is depicted on Figure 3.

There are three main ways to integrate biological data (Wanichthanarak et al. 2015). A common approach is to overlay multi‐omics data for pathway enrichment, which incorporates information about biochemical pathways or biological processes, typically using Gene Ontology databases for reference. Another approach is to perform biochemical network analysis, which draws connections between different types of cellular components to identify altered molecule neighbourhoods and connection hubs. An example of open‐source software for network analysis is MetScape (Karnovsky et al. 2012), a plug‐in for the Cytoscape software (Shannon et al. 2003). Lastly, when there is no biochemical domain knowledge, correlation analysis can be applied to integrate biological and other metadata, for instance clinical outcomes. The R package mixOmics, another open‐source software, provides useful statistical tools, such as sparse principal component analysis, canonical correlation analysis and sparse partial least squares (PLS) discriminant analysis (Rohart et al. 2017). It is important to realize that expression correlation and functional links between RNA, protein and metabolites in EVs are different from the links within cells. In general, if a mRNA is not present in a cell, the encoded protein and potential downstream metabolic conversions will not be present either. One cannot reach the same conclusion if a mRNA or the corresponding protein are missing from an EV population as they might be poorly loaded into EVs. Similarly, the correlation between enzyme expression levels, enzyme metabolic activity (level of active molecules) and their metabolic products is not a given in EVs. This phenomenon provides a challenge when utilizing cell omics integration and pathway analysis software with built‐in functional correlation analysis or predetermined pathway profiles. There is a clear need to generate specific EV transcriptome/proteome profiles for cell type deconvolution and adaptation of cell‐based functional omics‐integration software (Murillo et al. 2019; Nguyen et al. 2024; Li et al. 2020; Jeppesen et al. 2019; Hoshino et al. 2020). Investigators are starting to generate estimates of tissue‐ and cell‐type‐specific EV abundances in biological fluids for urine and blood derived EVs (Dwivedi et al. 2023; Larsen et al. 2024).

To implement the integration steps, many software tools have been developed, and a comprehensive list and considerations of which is most suitable for each focus can be found elsewhere (Jendoubi 2021; Pinu et al. 2019). Since Artificial Intelligence has emerged as an affordable tool, deep learning and specifically artificial neural networks such as variational auto‐encoders have been used to model multimodal data. Examples include Multigrate (Lotfollahi et al. 2022) and MultiVI (Ashuach et al. 2023), which apply unsupervised deep learning generative models to integrate multi‐omic modalities and provide low‐dimensional versions of each dataset. Replacing individual analyses with learnable embeddings can increase the interpretability of the model by comparing modality vectors (Lotfollahi et al. 2021).

In spite of these promising developments, there are a number of challenges in integration, including complex analysis pipelines for each omics dataset, assignment of biological information to genetic elements, intrinsic differences in the metabolome of individuals, and high internal correlation structures in the data (Jendoubi 2021). Importantly, these challenges are inherent to the methodology itself and do not only occur when applying EVs, rather represent general challenges for science. However, currently available commercial uEV platforms largely measure one type of analyte only (miR Sentinel Test, ExoDx prostate intelliscore (EPI) (Boehm et al. 2023)). It remains to be established whether the measurement of combinations of RNA, protein and metabolites at the same time from a single sample, will have sufficient added value in terms of biomarker performance to justify the commercial development of cost‐efficient integrated combi‐tests for uEV‐associated diseases. There is also the possibility to combine clinical information with novel biomarkers to strengthen the biomarker performance.

**FIGURE 4:**
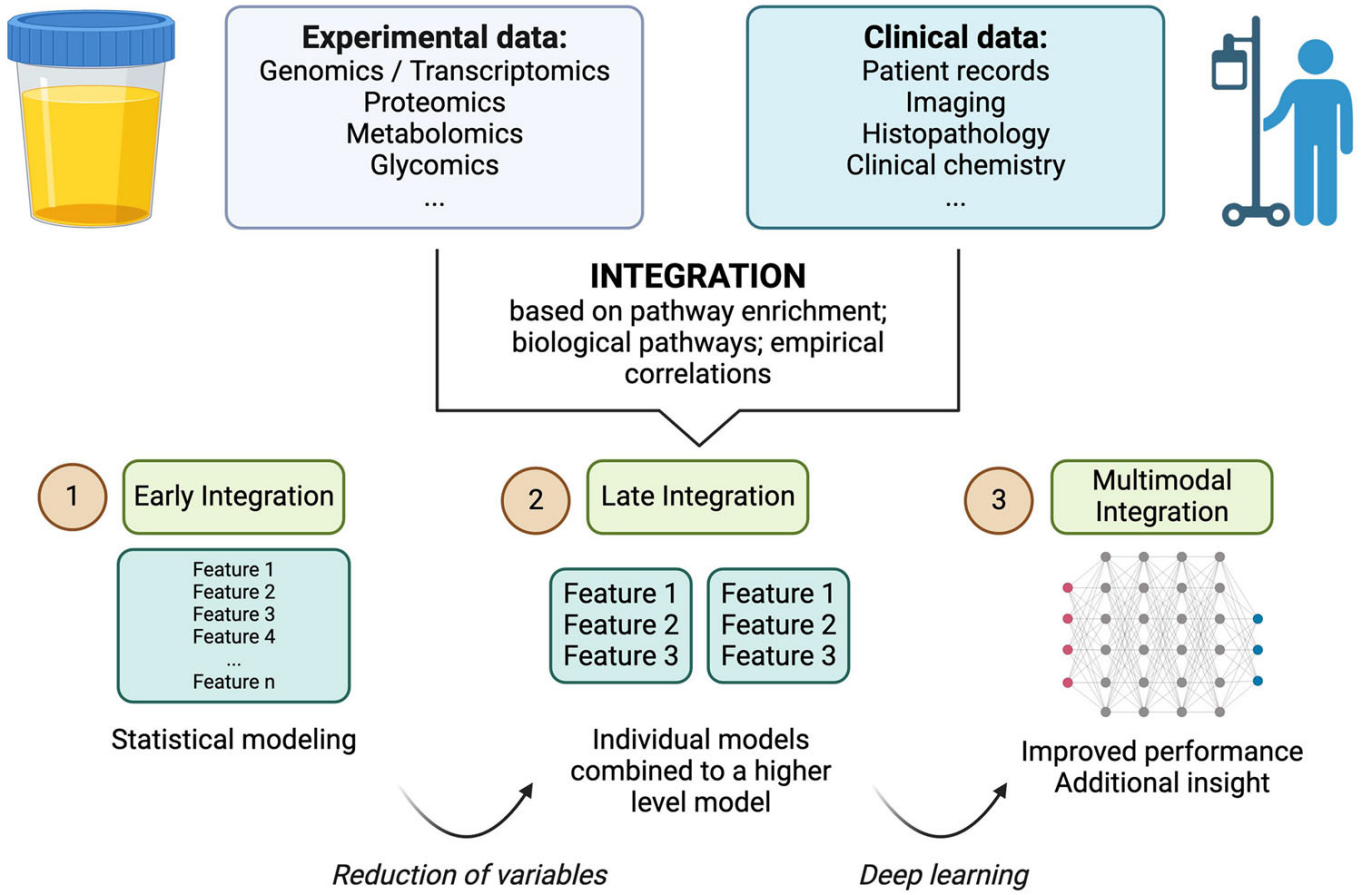
The process of designing the experimental and data integration pipeline involves making decisions about how data will be managed. One important choice is whether to integrate the data before or after reducing its dimensions, and before or after generating a model. The various approaches for integration are explained in the text, and the design should consider the specific goal to be achieved through integration, whether it is a sequential, biological, or model‐based objective. Figure created using BioRender.com.

To sum up, integration of omics data should be approached strategically and only pursued when it is expected to yield more informative results than a single analysis. Nevertheless, integration of EVs profiling with clinical parameters, biochemical analysis and individual genome data holds great promise for advancing disease modelling and its application in precision medicine (Figure 4).

## 5 Validation of uEV‐Based Biomarker Assays

Once developed, the next important step is uEV‐based biomarker assay validation before a test can be used in clinical research or in a diagnostic setting. Several factors should be considered, ranging from the intended use, turnaround time and validation strategy—for example, using pooled and/or individual samples, targeted sensitivity, specificity and reproducibility, disease prevalence and power calculations—and up to the final clinical utility and cost‐effectiveness (Paulovich et al. 2008; Parkinson et al. 2014; Marton and Weiner 2013). A validated assay may need to follow Good Laboratory Practice (GLP) and/or Good Clinical Practice (GCP) compliance, such as within toxicology studies and clinical trials. As only few uEV assays have been developed, literature examples or guides describing the development and validation of real‐life assays are scarce. Validation strategies have to be adopted mainly from other assays with similar analytes and may be suboptimal at first. Inventors should look for predicate approaches, but be prepared to be first in and design/set the standard.

**FIGURE 5:**
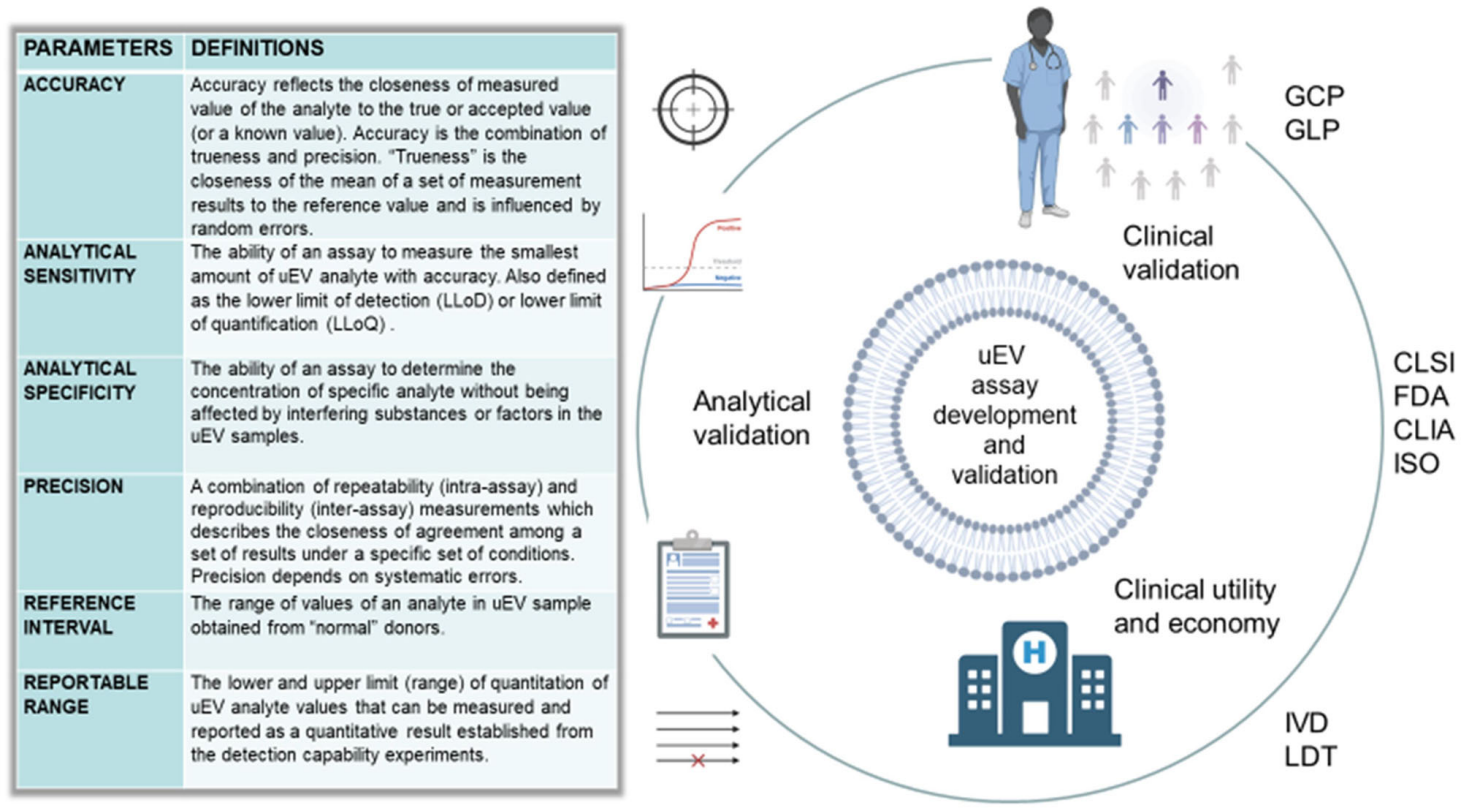
Schematic overview of requirement considerations in uEV assay development and validation. To meet the requirements of everyday clinical use, it has to be assured that the assay under development meets requirements of accuracy as well as analytical specificity and sensitivity. Moreover, reproducibility during repeated measurements and comparability of inter‐laboratory results have to be assured. For quantitative tests, reference intervals and reportable ranges must be determined separately, often in larger cohorts of healthy donors and patient target populations. GCP, Good Clinical Practice; GLP, Good Laboratory Practice; CLSI, Clinical and Laboratory Standards Institute; FDA, Food and Drug Administration; CLIA, Clinical Laboratory Improvement Amendments; ISO, International Organization for Standardization; IVD, In Vitro Diagnostics; LDT, Laboratory Developed Test. Figure created using BioRender.com.

Assay validation aims to demonstrate that the method is accurate, specific, reproducible and robust over a designated range of measurements. The main assay performance validation parameters include accuracy, analytical specificity and sensitivity, detection capability, precision, reference interval and reportable range (listed in Figure 5 with definitions). Measurement of assay performance requires reliable and scalable tools, analytical workflows and controls that could be missing for uEVs (Ayers et al. 2019). In addition, enough patient cohorts need to be recruited or access to large biobanks provided to validate the assay. The ExoDx Prostate Intelliscore (EPI) has done that in over 1000 patients and in the real‐world setting (McKiernan et al. 2016; McKiernan et al. 2018). I Finally, published guides and check lists may help to determine the options and requirements, commercial potential and technology readiness level (TRL) for the developed assay (Marton and Weiner 2013; Biomarker Commercialization Consortium (BIC)). It is important to understand who uses, chooses and pays for the technology (stakeholders), and who benefits from the technology (end users). There could be several reasons for not commercializing an uEV‐based assay, for example, a small market size due to a rare disease or analyte, a small profit margin due to the technical complexity of an assay, or the availability of alternative cheaper tests.

During validation, assay performance should be documented as recommended in the publications by the Clinical and Laboratory Standards Institute (CLSI) and International Organization for Standardization (ISO). In addition, well‐defined post‐validation calibration and quality control procedures should be outlined for performing maintenance. The various regulatory processes required and applicable to each assay development and life cycle phase—when aiming to translate the assay into a product—are summarized in ‘Technical Requirements and CLSI Guidelines for Laboratory Test Method Life Phases’ (local/CLIA/FDA/ISO; (United States Centers for Disease Control and Prevention 2021)). Evidently, navigating through the regulatory maze requires knowledge and resources that could not be immediately available to all. However, some academic institutions are starting to offer these resources by providing technology transfer services to their affiliates.

## 6 Approval Roadblocks

### 6.1 General Considerations on Bringing a Diagnostic Test to the Market

Two main paths to bring a diagnostic test to market are centralized testing (laboratory developed test or LDT in US, in house IVD or IH‐IVD in EU) or decentralized testing. These two models differ in legislation, logistics and operational requirements that involve different investments and time needed, scalability, profit opportunities. Another difference is the ability to control the key variables affecting test performance.

Overall robustness and ease‐to‐transfer are fundamental requisites that guide the choice of whether to launch a test as LDT or IVD. Independent of the regulatory model, a viable clinical grade diagnostic test must integrate the urine sample collection and processing (including transport and storage) with the EV analyte stability, extraction and downstream analytical platform and (as much as possible) in an operator independent manner. LDTs are used by a single central laboratory that relies on a sophisticated outreach network for controlled collection of patient specimens. Any issue can then be rapidly troubleshooted and resolved by the same lab staff that developed the test. An IVD test kit is a one‐size‐fits‐all solution; it must meet the needs of all customers and show robust interlaboratory consistency. For novel technologies, that often come without the method lock‐down (methods are still developed and improved) and low maturity of biomarkers, such as with EV‐based diagnostics, this is extremely challenging. However, if the implementation of a new technology does not disrupt the existing lab and clinical infrastructure, the chance of its ‘landing’ and adoption in the laboratories is much higher. Sometimes the IVD test must be performed on a particular instrument or piece of equipment, while other kits may be used on ‘open platform’ instruments that are test kit agnostic. Possibly, automated workflows, seamlessly integrable into state of art laboratory workstations would be lead candidates.

IVD providers undergo a lengthy and expensive process of regulatory clearance (United States Food and Drug Administration, FDA in US and European conformity mark, CE in EU). This investment by IVD manufacturers makes sense from the perspective of a larger volume of tests sold, when a strong market potential (i.e., profit) is envisioned. Therefore, some developers choose the LDT route (U.S. Food, and Drug Administration 2024) as a first step to generate both evidence and sales while heading for an IVD application (U.S. Food and Drug Administration 2021).

Although there has been a historical exemption of regulatory oversight for centralized laboratory testing, in the US the FDA maintains the authority to selectively regulate some high‐complexity methods and tests. Moreover, the changing regulatory landscape is creating uncertainties for reference laboratories both in US and EU. The Valid Act proposed placing LDTs and IVDs under the same FDA regulated scheme (Sauer‐Budge 2022). This was not accepted by the US Congress in 2022 but is expected to be reintroduced soon. In the EU, a new In Vitro Diagnostic Regulation (IVDR 2017/746) entered in application in May 2022, significantly tightening the requirements for IVD designation and monitoring of Notified Bodies by augmenting the technical documentation and clinical evidence needed. IVDR primarily regulates commercially sold CE‐IVDs, but also addresses in‐house IVDs, that are manufactured, modified and used by health institutions in a nonindustrial scale to meet the specific needs of target patient groups if no equivalent CE marked device is available. Regulatory trends toward use of harmonized standards and international guidelines bring the diagnostics approval and marketing in Europe and US in a closer alignment. However, FDA approval remains significantly more expensive, with a review cycle about three times longer and requiring full clinical trials, whereas the CE‐Mark can still be obtained through a clinical evaluation of published data for equivalent devices. A post‐marketing follow‐up study is still requested for CE marked tests and devices but remains less arduous than the requirements for a de novo FDA application. In addition, CE‐Mark is recognized almost globally, which makes it more attractive to many diagnostic companies to apply for the CE‐Mark first.

**FIGURE 6:**
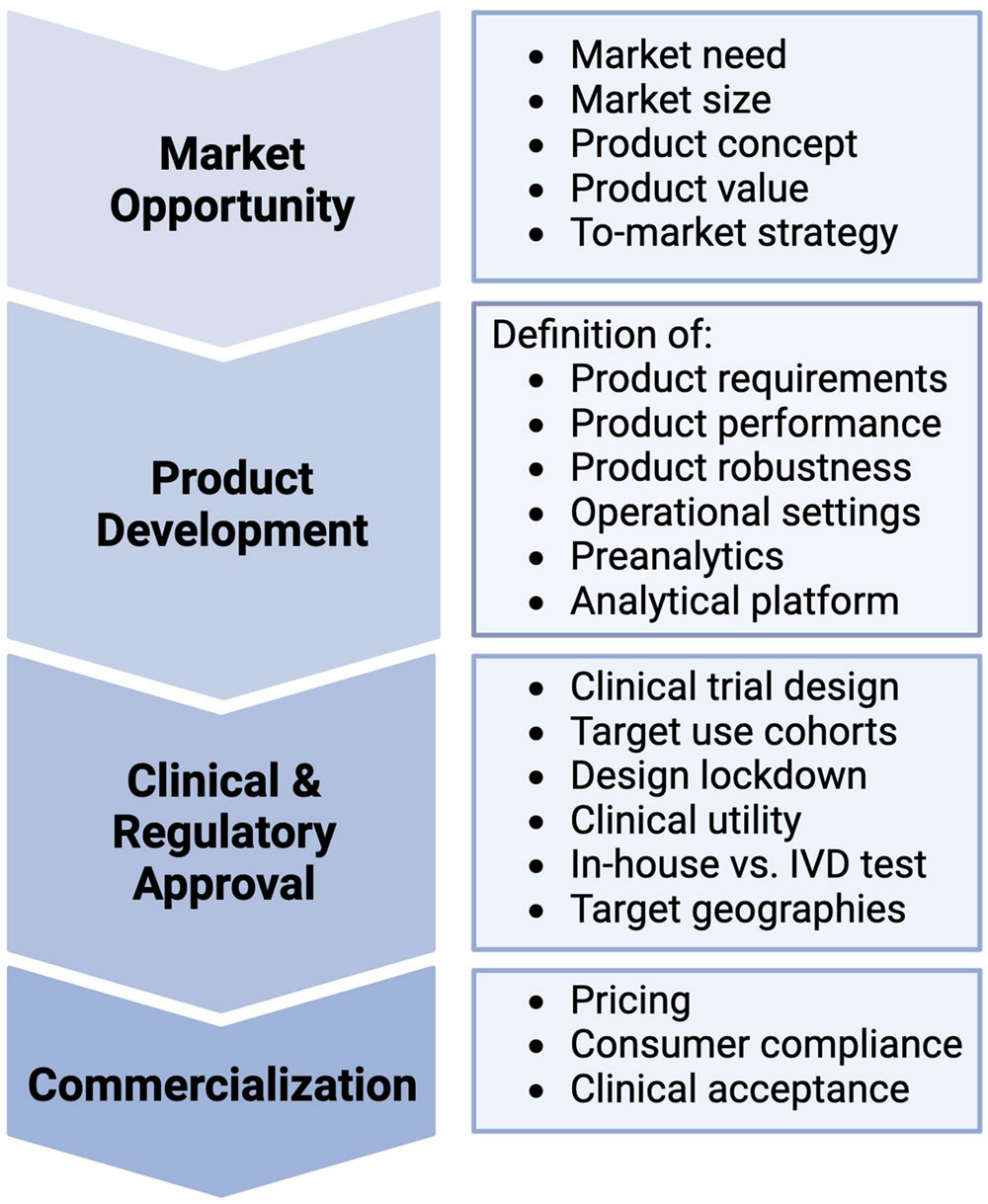
Simplified to‐market roadmap, including the regulatory process. Regulatory processes should already be considered from the early phases of diagnostic product development. This assures that the envisioned product passes approval in later phases. Several strategic decisions have to be made: For instance, whether the proposed test should be marketed as an IVD or LDT in‐house assay can severely impact the requirements regarding simplicity of protocols and need of specialized technology. During the clinical validation process, the developing entity (e.g., an academic institution or a company) typically works together with clinical experts to design meaningful clinical trials and conduct them in a GCP‐compliant manner. The partnership with clinicians is also very important during implementation of the new diagnostic test in clinical routine, especially during collecting and reporting of evidence needed to recommend its use in clinical guidelines. Figure created using BioRender.com.

In seeking a path‐to‐approval and market of a novel diagnostic test one may consider following regulatory precedents. The only marketed EV diagnostic solution to‐date is Prostate IntelliScore (EPI) test based on urinary EV mRNA signature developed by Exosome Diagnostics (BioTechne). This was the first EV‐based liquid biopsy test to receive the FDA Breakthrough Device Designation in 2019, and an IVD‐CE Mark in 2021. In both territories, it is marketed through a centralized lab facility. The development of this test, encompassing the four biomarker discovery cohorts (100+ patients each), feasibility study for signature lock down (200–500 patients), two independent validation cohorts (500+ patients) and clinical utility study (1000+ patients) took 7 years, and tens of millions dollar investment (Tutrone et al. 2020). A detailed overview of the key landmarks in the development journey of such a test can be found in the Supporting Information; a simplified to‐market roadmap is provided in Figure 6.

## 7 Clinical Roadblocks

It is tempting to view regulatory approval as the major hurdle for introducing a novel diagnostic test into the health care setting. However, regulatory approval rarely takes into account clinical implementation and uptake; critical determinants of whether a diagnostic test will enter routine clinical management. If the test is to be employed in a decentralized manner, then establishment of adequate infrastructure in target labs is fundamental to establishing a uEV‐based clinical test. At present, most EV‐based analyses employ sophisticated equipment that is not always available in laboratories. Even if this hurdle can be overcome, appropriate training for those conducting the test is necessary to ensure validity (such as for any novel medical device). Issues related to training and assay protocols are consistently noted as barriers to implementation of new clinical assays (Pai et al. 2015; Ivanov 2013). Extending from this is the issue of the cost of implementation. There is a clear up‐front cost for acquisition of instrumentation and training of staff, and ongoing ‘per test’ costs may be a barrier. This is particularly relevant in regions such as the United States where private health insurers decide whether a new diagnostic test is covered (Ivanov 2013; Billings 2006). Interestingly, the perceived cost of an assay may be more of a barrier than its actual cost. A recent study noted that more than 80% of health care practitioners believe that the costs of in vitro diagnostic tests are higher than they actually are (Rohr et al. 2016). One approach to overcoming these barriers is to develop a centralized LDT/IH‐IVD as has been done for the EPI test mentioned above. However, while this may overcome cost and infrastructure barriers, it may not be appropriate for clinical tests where immediacy of result is the priority.

Clinical inertia has also been consistently identified as a barrier to implementation of new tests and therapies (Pai et al. 2015; Ivanov 2013; Billings 2006). In this regard clinicians may express scepticism about whether uEV approaches are superior to those already available and be reluctant to replace established methodology. There may also be concerns about impact on clinical workflows: that a new test will increase unnecessary testing or an over‐reliance could undermine clinical skills. Efforts to educate clinicians on the value of a new test and to demonstrate real world utility and improved performance compared to routine tests are likely to overcome clinical inertia but this may take time.

Another important consideration is that clinical landscapes can also change rapidly. A gap served by a diagnostic test under development can theoretically already be served by another, cheaper or more advantageous alternative, as the development phase usually extends over years (sometimes decades). One example of this is the development of urine biomarkers for prostate cancer, which should overcome the inaccuracies of the widely used PSA test. The market for such urine tests has changed with the development of multiparametric MRI of the prostate, which, in addition to greater diagnostic accuracy compared to PSA, also offers the possibility of targeted fusion biopsy of conspicuous lesions and is relatively inexpensive due to its widespread availability (Saltman et al. 2021). This makes it clear that once a test has reached market maturity, it is a constant task to critically re‐evaluate suitable clinical fields of its application and, if necessary, to open up new ones through appropriate clinical studies.

**FIGURE 7:**
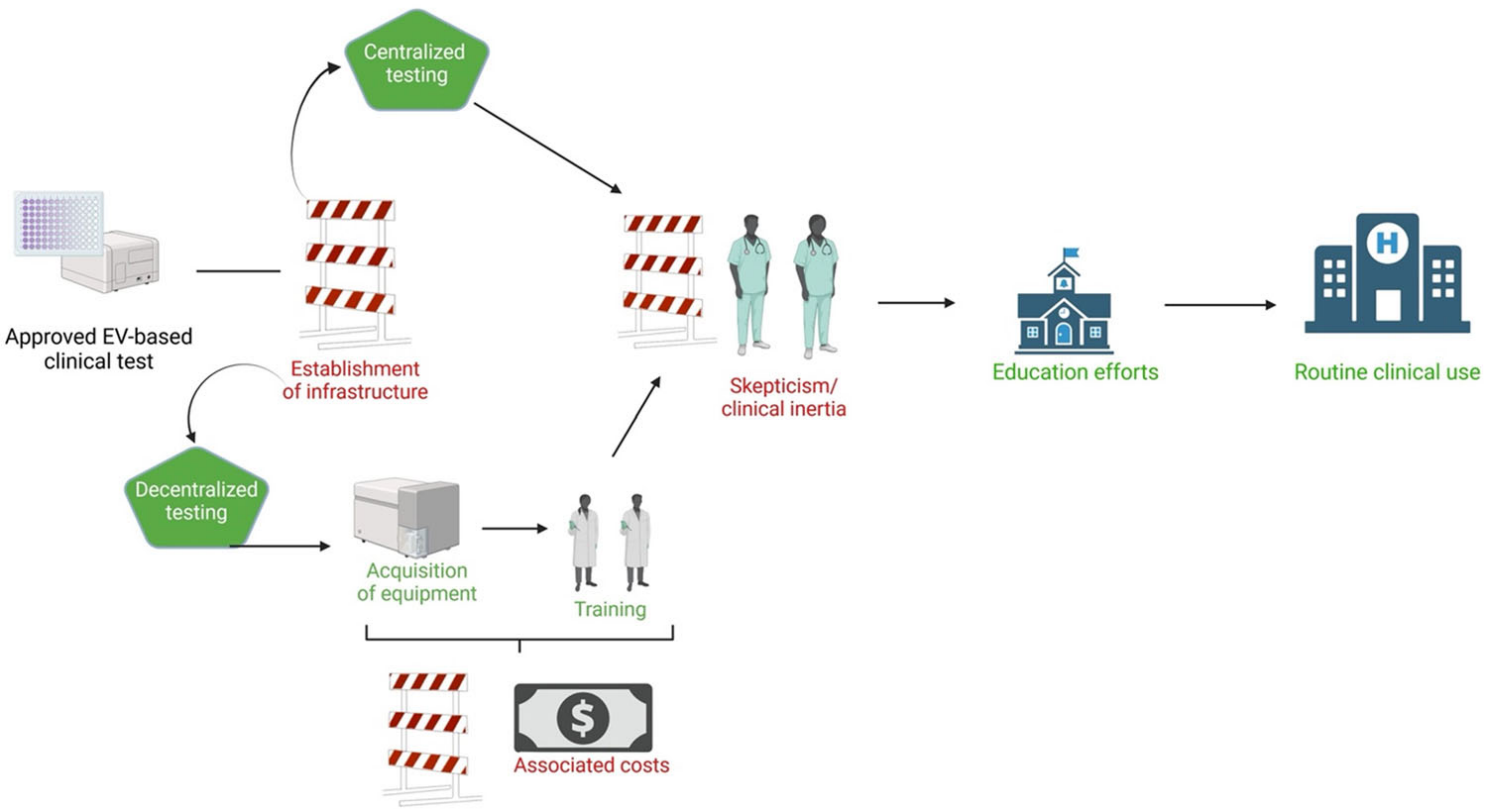
Clinical roadblocks of uEV biomarker translation. Once a novel test has completed regulatory approval, several hurdles may occur on the path toward routine clinical use. Depending on the product, additional costs for equipment and training may have to be covered, especially for IVD tests that are offered for decentralized testing. Regardless of the mode of distribution, novel tests often face clinical inertia or even scepticism, since a benefit compared to established standards of care must be scientifically (and often economically) demonstrated and accepted by physicians—which is, for instance, facilitated by incorporating the test into clinical guidelines. This, in turn, requires long‐term educational efforts within the specific field.Figure created using BioRender.com.

The various mentioned roadblocks in the route to clinical acceptance are depicted in Figure 7.

## 8 The Road Ahead and the Light at the End of the Tunnel

Knowledge about urinary EVs has progressed exponentially since small uEVs were first described (Wiggins et al. 1986). The possible strengths of using uEVs for diagnostics have been widely discussed in the literature: uEV cargo is protected by a lipid bilayer which makes it more stable compared to other cell‐free molecules and less susceptible to catabolic enzymes and are small enough to cross cellular barriers (Nik Mohamed Kamal and Shahidan 2019). Moreover, uEVs can be released as a part of physiological cell‐to‐cell communication which enables uEV‐based tests to directly target disease‐causing molecules instead of purely being surrogate markers of disease activity (Giuliani et al. 2022). Overall, uEVs offer a non‐invasive approach to gain insight into cell homeostasis that might ultimately replace or complement invasive testing such as percutaneous biopsies.

Here, we discussed the major roadblocks to clinical translation of uEV biomarkers, but also offer possible solutions to overcome them. Many of the outlined barriers can be tackled with the undertaking of in‐depth physiological studies of uEV biology, the standardization of EV collection, separation and characterization and the improvement of technologies for single EV analysis. Assay development, validation and approval should be augmented by clear goal setting, involvement of clinicians and counselling the regulatory authorities early in the assay development. Clinical roadblocks can be subsequently overcome when reliable assays address unsolved bottlenecks in clinical decision making and can be integrated into existing clinical pathways. The ultimate goal is to develop standardized, robust methods that offer rapid analytical platforms compared to the current time‐consuming EV enrichment and separation methodologies. Novel technologies should be capable of fast, ultrasensitive detection and have the ability to capture multiple signals in clinically available samples simultaneously and in real time, ideally by label‐free approaches.

Currently, similar to other emerging precision medicine strategies, uEV diagnostics are in a position comparable to genetic testing 30 years ago—we know uEVs exist and hold great potential, however, we have yet to discover the uEV cargoes that are most indicative of disease states and the methodologies that are most effective to measure and quantify it. To achieve and improve translation of uEV diagnostics many national EV societies and in particular the ISEV lead multi‐focal endeavours to promote reproducibility, foster collaboration and develop research infrastructure:

1. **Rigor and standardization**—ISEV's rigor and standardisation subcommittee has the goal of improving reproducibility on EV research. Beside the Minimum Information for Studies of EVs (MISEV) guidelines to standardise reporting of EV studies (Lötvall et al. 2014) the urine task force has provided a position statement on urinary EVs (Erdbrügger et al. 2021) and more recently a guide for the collection of uEVs and a quick reference card (van Royen et al. 2023).
2. **Network science—**Clinical implementation of uEVs requires multi‐disciplinary teams across academia, industry and healthcare. Analogous to public sequencing databases, EV‐TRACK is a crowdsourcing knowledgebase (http://evtrack.org) which centralises research methodologies and results (Van Deun et al. 2017). Task forces for implementing Rigor and Reproducibility and Special interest groups foster research networks across scientific disciplines such as nanotechnology, cellular biology and clinical sciences. Additionally, network meetings and workshops such as the recent ISEV workshop ‘Extracellular Vesicle (EV)‐based biomarkers: Commercialisation and Clinical Implementation’ (https://www.isev.org/ev‐based‐biomarkers) help to discuss overarching roadblocks and to form cross‐institutional initiatives to target them.
3. **Raising EV awareness**—Special interest groups and workshops, also within ISEV, organize numerous educational activities to increase awareness of EV biology and methodology beyond EV scientists. Evolved from the ISEV Rigor and Standardization Urine Task Force, the Genitourinary EV (GUSEV) special interest group have held online seminars, journal clubs and cross‐society meeting sessions (e.g., European Society of Urological Research and ISEV) to target kidney, urological and reproductive system researchers (International Society for Extracellular Vesicles 2023).

With these tools in play, the future of uEV translation into clinical practice is possible, the foundation is already well developed and thriving. The collaborative effort between scientists, clinicians, engineers, industry and regulatory institutions can move this promising field forward and realize its full potential.

## Author Contributions

**Marvin Droste**: Conceptualization (lead); data curation (lead); investigation (equal); project administration (lead); resources (lead); software (lead); supervision (supporting); visualization (lead); writing ‐ original draft (lead); writing ‐ review and editing (lead). **Maija Puhka**: Conceptualization (supporting); investigation (equal); visualization (equal); writing ‐ original draft (equal); writing ‐ review and editing (equal). **Charles Blijdorp**: Investigation (equal); writing ‐ original draft (equal); writing ‐ review and editing (supporting). **Gloria Alvarez‐Llamas**: Investigation (equal); writing ‐ original draft (equal); writing ‐ review and editing (supporting). **Benedetta Bussolati**: Investigation (equal); writing ‐ original draft (equal); writing ‐ review and editing (supporting). **James W. Dear**: Investigation (equal); writing ‐ original draft (equal); writing ‐ review and editing (supporting). **Bernd Giebel**: Investigation (equal); writing ‐ original draft (equal); writing ‐ review and editing (supporting). **Cristina Grange**: Investigation (equal); writing ‐ original draft (equal); writing ‐ review and editing (supporting). **Janne Leivo**: Investigation (equal); writing ‐ original draft (equal); writing ‐ review and editing (supporting). **Metka Lenassi**: Writing ‐ review and editing (supporting). **Alicia Llorente**: Investigation (equal); writing ‐ original draft (equal); writing ‐ review and editing (supporting). **Inge Mertens**: Investigation (equal); writing ‐ original draft (equal). **Harald Mischak**: Investigation (equal); writing ‐ original draft (equal); writing ‐ review and editing (supporting). **Desmond Pink**: Investigation (equal); writing ‐ original draft (equal); writing ‐ review and editing (supporting). **Tobias Tertel**: Investigation (equal); writing ‐ original draft (equal); writing ‐ review and editing (supporting). **Swasti Tiwari**: Investigation (equal); visualization (equal); writing ‐ original draft (equal); writing ‐ review and editing (supporting). **Dolores Di Vizio**: Writing ‐ review and editing (supporting). **Natasa Zarovni**: Investigation (equal); visualization (equal); writing ‐ original draft (equal); writing ‐ review and editing (supporting). **Guido Jenster**: Conceptualization (supporting); investigation (equal); writing ‐ original draft (equal); writing ‐ review and editing (equal). **Dylan Burger**: Conceptualization (supporting); investigation (equal); visualization (equal); writing ‐ original draft (equal); writing ‐ review and editing (equal). **Uta Erdbrügger**: Conceptualization (lead); data curation (supporting); funding acquisition (lead); investigation (equal); project administration (lead); resources (lead); supervision (lead); visualization (equal); writing ‐ original draft (lead); writing ‐ review and editing (lead).

## Conflicts of Interest

Desmond Pink is the founder and employee at Nanostics Inc. Fabrice Lucien discloses research funding and financial compensation from NaNotics LLC, licensing agreement and royalties from Early Is Good and consulting for Mursla Bio. Marvin Droste discloses speaking fees from Novartis, outside of the published work. All other authors report no conflict of interest.

## Supporting information




**Supplementary Table**: jev270120‐sup‐0001‐TableS1.docx

## Data Availability

Data sharing is not applicable to this article as no datasets were generated or analysed during the current study
