# Supplementary material for: Roadblocks of Urinary EV Biomarkers: Moving Toward the Clinic
Source: J Extracell Vesicles. 2025 Jul 17;14(7):e70120. doi: 10.1002/jev2.70120 (PMC12269535; doi:10.1002/jev2.70120)
Supplement: Supplementary file 1 — Supplementary Table: jev270120‐sup‐0001‐TableS1.docx [file JEV2-14-e70120-s001.docx]

**Supplementary Material**

| Landmark | Premises | Actions |
| --- | --- | --- |
| Clear Regulatory and Marketing Path | Dependent on indication of use and test classification, overall timeline and the resources availability (funding), market prospect and test operative robustness | US: LDT, clinical laboratories are regulated by the Centers for Medicare and Medicaid Services (CMS) with authority through the Clinical Laboratory Improvement Amendments (CLIA). Drug Administration (FDA) actively regulates some laboratory tests. IVDs must be approved by the FDA prior to selling via 510(k) or Pre-market approval (PMA) pathway.  EU: Hospitals can run IH-IVD without CE-Mark if no equivalent CE-IVD exists but will need appropriate ISO certification (15189) Novel IVDR foresees CE marking of all commercial diagnostic tests with the active role of notified bodies |
| Validated, feasible, controlable, preanalytical SOPs | Preanalytical variables account for 32-75% of lab errors. A viable clinical diagnostic test must smoothly integrate the urine collection transport and storage. | All the elements of diagnostic flow need to be optimized and certified – from the urine collection device or cup, through all the reagents and controls. LDTs control variables by adopting sophisticated devices and network for sample collection. The robustness of SOPs for IVDs is best checked when one shifts to multicentric trial design or launches testing in independent sites. |
| High sensitivity and wide dynamic range | The sensitivity for detection of rare EV born molecules, is often critical for given indication of use | Disease related EVs and associated markers are heavily outnumbered by other biofluid components, including irrelevant EVs, lipoproteins, circulating molecules. E.g., EPI test has LOD of 10 RNA molecules and spans over five orders of magnitude |
| Appropriate design of validation trials | The cohorts must match the targeted indication/population. Clinical utility is a key driver for approval and adoption. | This can be challenging and expensive if test developers are seeking FDA approval, which often requires an ethnically diverse cohort for certain tests. The size of trial matters, typically comprising 1,000-3,000 patients enrolled in a registration trial |
| Assured clinical grade robustness | The robustness must be assured across all the steps, assuring reproducible clinical EV preparations, extraction phase and analytical processing. | The goal is to bring down the SD of the test to that one would expect from the used method itself QC of all reagent batches needs to be routinely implemented, with reproducibility testing across of 100s of technical replicates and possibly 1000s of clinical samples. |
| Assured transability of biomarker panels | The continuity/coherence is needed across whole biomarker discovery & validation process | It is highly recommendable to use in all the phases of development the same methodology and sample matrix that will be used for a final diagnostic scenario. |
| Defined indication of use | The formulation of all the possible indications and claims orients the whole technical and business development. | To do this early, intended consumers should be interrogated early, in a pre-design phase, and involve them, via voice of key opinion leaders (KOLs), at each step of the involvement (for instance formulation of diagnostic cut-offs). |
| Establish payment and reimbursement | The pricing strategy fundamentally linked with indication of use, clinical utility and product specifications. | The price will determine the line of diagnostics it will go for – screening or confirmatory. EPI test costs around $750, with respect to $500 for MRI, $1300 for a biopsy, or $50 for PSA. Differentiators that support premium pricing, and *vice versa* need to be clear and anticipated. |

**Table S1: Key landmarks of the to-market journey of a novel diagnostic test.**
